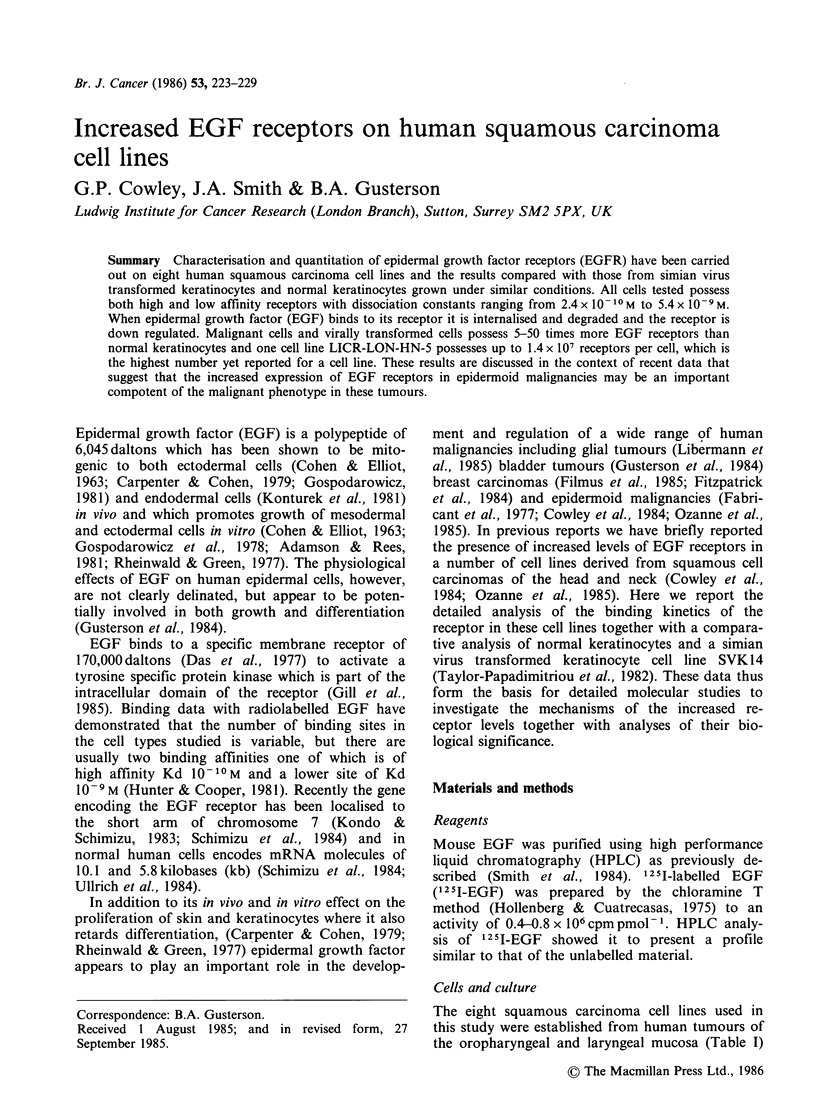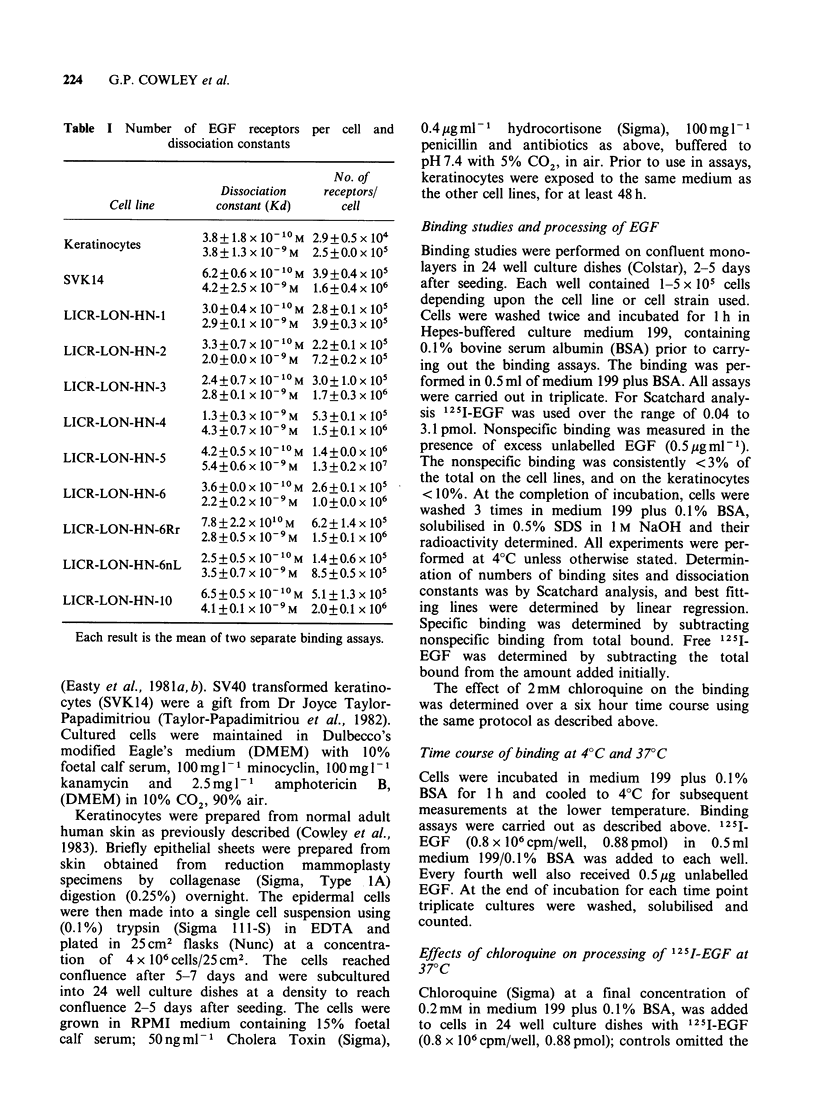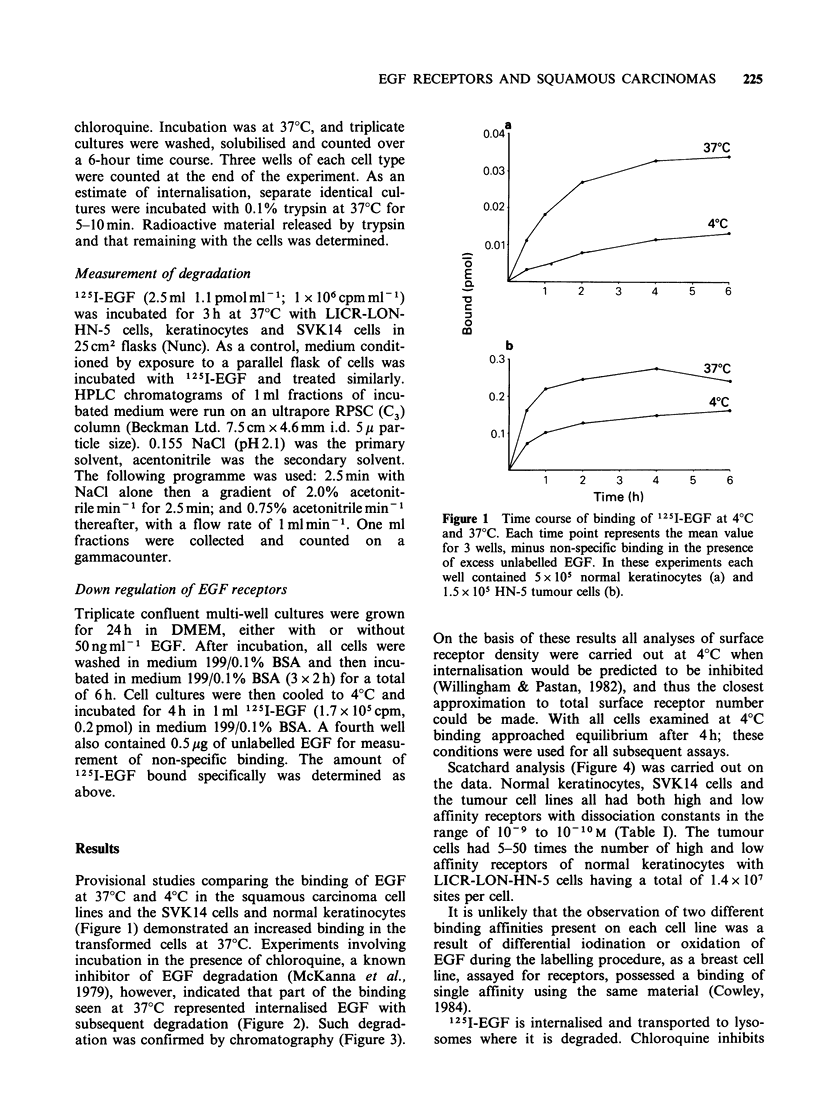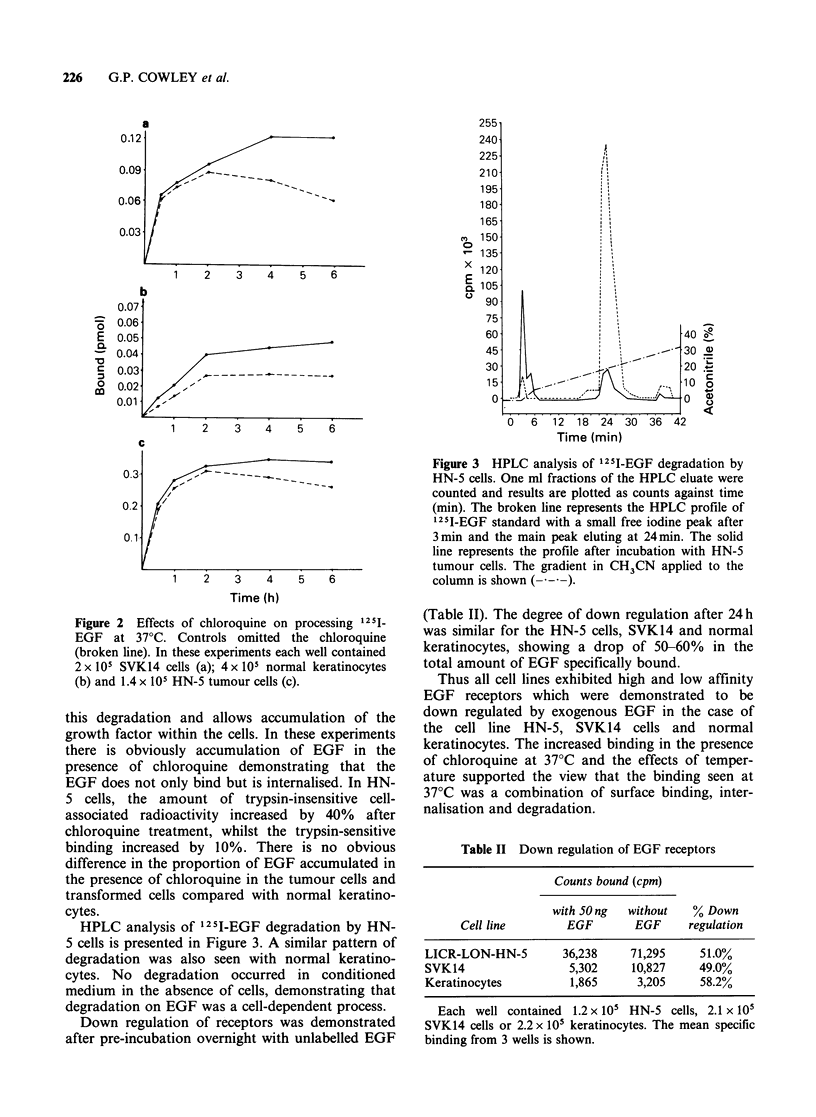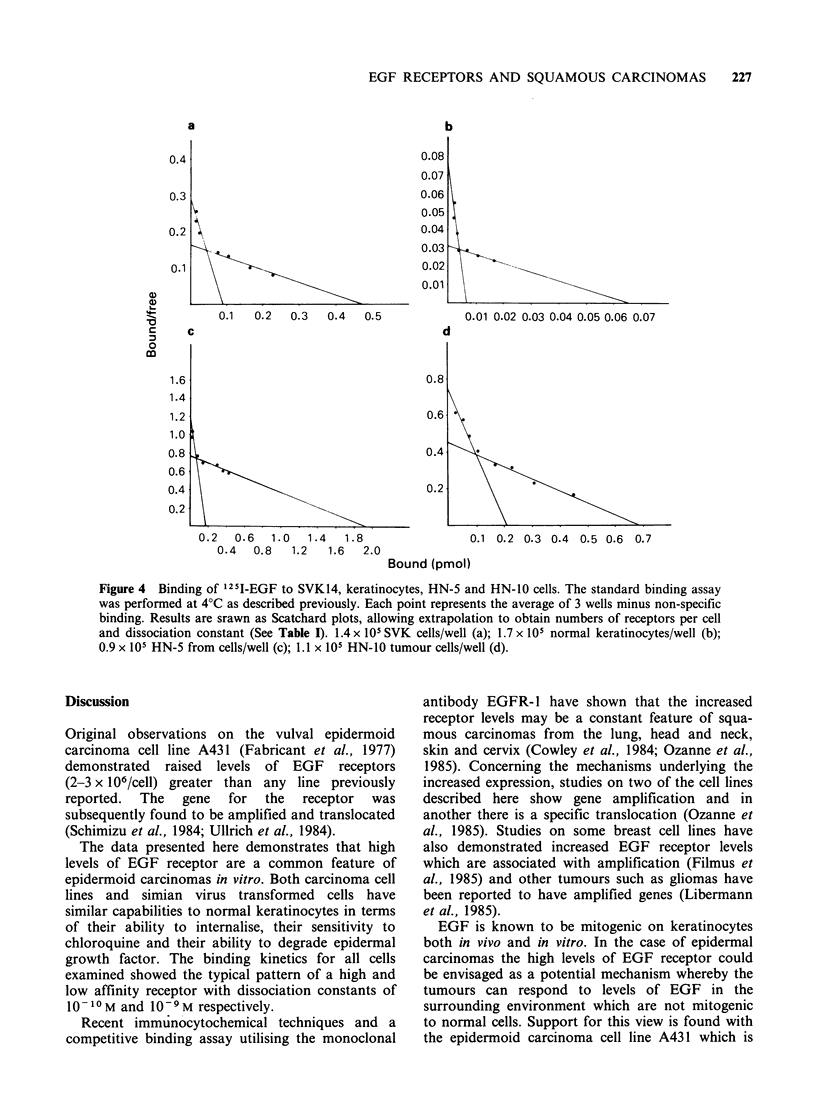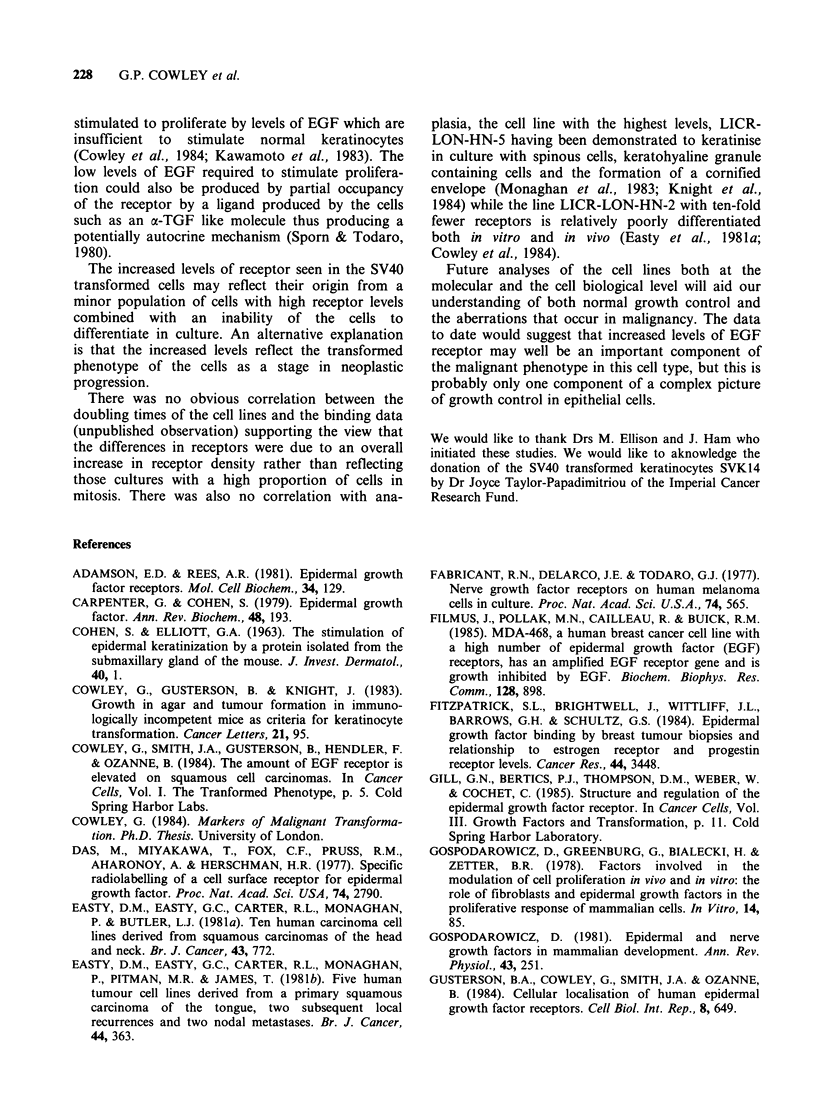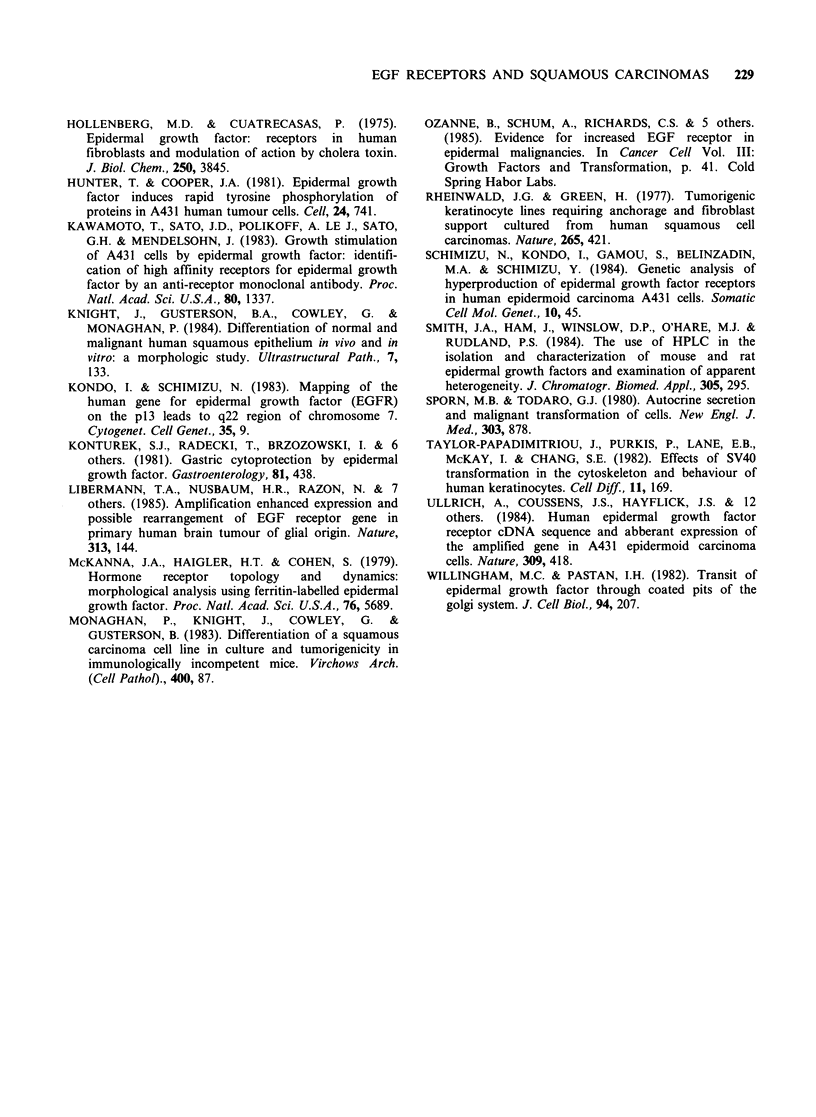# Increased EGF receptors on human squamous carcinoma cell lines.

**DOI:** 10.1038/bjc.1986.39

**Published:** 1986-02

**Authors:** G. P. Cowley, J. A. Smith, B. A. Gusterson

## Abstract

Characterisation and quantitation of epidermal growth factor receptors (EGFR) have been carried out on eight human squamous carcinoma cell lines and the results compared with those from simian virus transformed keratinocytes and normal keratinocytes grown under similar conditions. All cells tested possess both high and low affinity receptors with dissociation constants ranging from 2.4 X 10(-10) M to 5.4 X 10(-9) M. When epidermal growth factor (EGF) binds to its receptor it is internalised and degraded and the receptor is down regulated. Malignant cells and virally transformed cells possess 5-50 times more EGF receptors than normal keratinocytes and one cell line LICR-LON-HN-5 possesses up to 1.4 X 10(7) receptors per cell, which is the highest number yet reported for a cell line. These results are discussed in the context of recent data that suggest that the increased expression of EGF receptors in epidermoid malignancies may be an important component of the malignant phenotype in these tumours.


*Br. J. Cancer* (1986) **53**, 223–229

### Increased EGF receptors on human squamous carcinoma cell lines

G.P. Cowley, J.A. Smith & B.A. Gusterson

*Ludwig Institute for Cancer Research (London Branch), Sutton, Surrey SM2 5PX, UK*

**Summary** Characterisation and quantitation of epidermal growth factor receptors (EGFR) have been carried out on eight human squamous carcinoma cell lines and the results compared with those from simian virus transformed keratinocytes and normal keratinocytes grown under similar conditions. All cells tested possess both high and low affinity receptors with dissociation constants ranging from $2.4 \times 10^{-10}$ M to $5.4 \times 10^{-9}$ M. When epidermal growth factor (EGF) binds to its receptor it is internalised and degraded and the receptor is down regulated. Malignant cells and virally transformed cells possess 5–50 times more EGF receptors than normal keratinocytes and one cell line LICR-LON-HN-5 possesses up to $1.4 \times 10^7$ receptors per cell, which is the highest number yet reported for a cell line. These results are discussed in the context of recent data that suggest that the increased expression of EGF receptors in epidermoid malignancies may be an important compotent of the malignant phenotype in these tumours.

Epidermal growth factor (EGF) is a polypeptide of 6,045 daltons which has been shown to be mitogenic to both ectodermal cells (Cohen & Elliot, 1963; Carpenter & Cohen, 1979; Gospodarowicz, 1981) and endodermal cells (Konturek *et al.*, 1981) *in vivo* and which promotes growth of mesodermal and ectodermal cells *in vitro* (Cohen & Elliot, 1963; Gospodarowicz *et al.*, 1978; Adamson & Rees, 1981; Rheinwald & Green, 1977). The physiological effects of EGF on human epidermal cells, however, are not clearly delinated, but appear to be potentially involved in both growth and differentiation (Gusterson *et al.*, 1984).

EGF binds to a specific membrane receptor of 170,000 daltons (Das *et al.*, 1977) to activate a tyrosine specific protein kinase which is part of the intracellular domain of the receptor (Gill *et al.*, 1985). Binding data with radiolabelled EGF have demonstrated that the number of binding sites in the cell types studied is variable, but there are usually two binding affinities one of which is of high affinity Kd $10^{-10}$ M and a lower site of Kd $10^{-9}$ M (Hunter & Cooper, 1981). Recently the gene encoding the EGF receptor has been localised to the short arm of chromosome 7 (Kondo & Schimizu, 1983; Schimizu *et al.*, 1984) and in normal human cells encodes mRNA molecules of 10.1 and 5.8 kilobases (kb) (Schimizu *et al.*, 1984; Ullrich *et al.*, 1984).

In addition to its *in vivo* and *in vitro* effect on the proliferation of skin and keratinocytes where it also retards differentiation, (Carpenter & Cohen, 1979; Rheinwald & Green, 1977) epidermal growth factor appears to play an important role in the development and regulation of a wide range of human malignancies including glial tumours (Libermann *et al.*, 1985) bladder tumours (Gusterson *et al.*, 1984) breast carcinomas (Filmus *et al.*, 1985; Fitzpatrick *et al.*, 1984) and epidermoid malignancies (Fabricant *et al.*, 1977; Cowley *et al.*, 1984; Ozanne *et al.*, 1985). In previous reports we have briefly reported the presence of increased levels of EGF receptors in a number of cell lines derived from squamous cell carcinomas of the head and neck (Cowley *et al.*, 1984; Ozanne *et al.*, 1985). Here we report the detailed analysis of the binding kinetics of the receptor in these cell lines together with a comparative analysis of normal keratinocytes and a simian virus transformed keratinocyte cell line SVK14 (Taylor-Papadimitriou *et al.*, 1982). These data thus form the basis for detailed molecular studies to investigate the mechanisms of the increased receptor levels together with analyses of their biological significance.

Correspondence: B.A. Gusterson.
Received 1 August 1985; and in revised form, 27 September 1985.

### Materials and methods

### *Reagents*

Mouse EGF was purified using high performance liquid chromatography (HPLC) as previously described (Smith *et al.*, 1984). $^{125}$I-labelled EGF ($^{125}$I-EGF) was prepared by the chloramine T method (Hollenberg & Cuatrecasas, 1975) to an activity of $0.4$–$0.8 \times 10^6$ cpm pmol$^{-1}$. HPLC analysis of $^{125}$I-EGF showed it to present a profile similar to that of the unlabelled material.

### *Cells and culture*

The eight squamous carcinoma cell lines used in this study were established from human tumours of the oropharyngeal and laryngeal mucosa (Table I)

© The Macmillan Press Ltd., 1986

**Table I** Number of EGF receptors per cell and dissociation constants

| *Cell line* | *Dissociation constant (Kd)* | *No. of receptors/ cell* |
|---|---|---|
| Keratinocytes | $3.8 \pm 1.8 \times 10^{-10}$ M | $2.9 \pm 0.5 \times 10^{4}$ |
| | $3.8 \pm 1.3 \times 10^{-9}$ M | $2.5 \pm 0.0 \times 10^{5}$ |
| SVK14 | $6.2 \pm 0.6 \times 10^{-10}$ M | $3.9 \pm 0.4 \times 10^{5}$ |
| | $4.2 \pm 2.5 \times 10^{-9}$ M | $1.6 \pm 0.4 \times 10^{6}$ |
| LICR-LON-HN-1 | $3.0 \pm 0.4 \times 10^{-10}$ M | $2.8 \pm 0.1 \times 10^{5}$ |
| | $2.9 \pm 0.1 \times 10^{-9}$ M | $3.9 \pm 0.3 \times 10^{5}$ |
| LICR-LON-HN-2 | $3.3 \pm 0.7 \times 10^{-10}$ M | $2.2 \pm 0.1 \times 10^{5}$ |
| | $2.0 \pm 0.0 \times 10^{-9}$ M | $7.2 \pm 0.2 \times 10^{5}$ |
| LICR-LON-HN-3 | $2.4 \pm 0.7 \times 10^{-10}$ M | $3.0 \pm 1.0 \times 10^{5}$ |
| | $2.8 \pm 0.1 \times 10^{-9}$ M | $1.7 \pm 0.3 \times 10^{6}$ |
| LICR-LON-HN-4 | $1.3 \pm 0.3 \times 10^{-9}$ M | $5.3 \pm 0.1 \times 10^{5}$ |
| | $4.3 \pm 0.7 \times 10^{-9}$ M | $1.5 \pm 0.1 \times 10^{6}$ |
| LICR-LON-HN-5 | $4.2 \pm 0.5 \times 10^{-10}$ M | $1.4 \pm 0.0 \times 10^{6}$ |
| | $5.4 \pm 0.6 \times 10^{-9}$ M | $1.3 \pm 0.2 \times 10^{7}$ |
| LICR-LON-HN-6 | $3.6 \pm 0.0 \times 10^{-10}$ M | $2.6 \pm 0.1 \times 10^{5}$ |
| | $2.2 \pm 0.2 \times 10^{-9}$ M | $1.0 \pm 0.0 \times 10^{6}$ |
| LICR-LON-HN-6Rr | $7.8 \pm 2.2 \times 10^{10}$ M | $6.2 \pm 1.4 \times 10^{5}$ |
| | $2.8 \pm 0.5 \times 10^{-9}$ M | $1.5 \pm 0.1 \times 10^{6}$ |
| LICR-LON-HN-6nL | $2.5 \pm 0.5 \times 10^{-10}$ M | $1.4 \pm 0.6 \times 10^{5}$ |
| | $3.5 \pm 0.7 \times 10^{-9}$ M | $8.5 \pm 0.5 \times 10^{5}$ |
| LICR-LON-HN-10 | $6.5 \pm 0.5 \times 10^{-10}$ M | $5.1 \pm 1.3 \times 10^{5}$ |
| | $4.1 \pm 0.1 \times 10^{-9}$ M | $2.0 \pm 0.1 \times 10^{6}$ |

Each result is the mean of two separate binding assays.

(Easty *et al.*, 1981*a*,*b*). SV40 transformed keratinocytes (SVK14) were a gift from Dr Joyce Taylor-Papadimitriou (Taylor-Papadimitriou *et al.*, 1982). Cultured cells were maintained in Dulbecco's modified Eagle's medium (DMEM) with 10% foetal calf serum, $100\,mg\,l^{-1}$ minocyclin, $100\,mg\,l^{-1}$ kanamycin and $2.5\,mg\,l^{-1}$ amphotericin B, (DMEM) in 10% $CO_2$, 90% air.

Keratinocytes were prepared from normal adult human skin as previously described (Cowley *et al.*, 1983). Briefly epithelial sheets were prepared from skin obtained from reduction mammoplasty specimens by collagenase (Sigma, Type 1A) digestion (0.25%) overnight. The epidermal cells were then made into a single cell suspension using (0.1%) trypsin (Sigma 111-S) in EDTA and plated in $25\,cm^2$ flasks (Nunc) at a concentration of $4 \times 10^6$ cells/$25\,cm^2$. The cells reached confluence after 5–7 days and were subcultured into 24 well culture dishes at a density to reach confluence 2–5 days after seeding. The cells were grown in RPMI medium containing 15% foetal calf serum; $50\,ng\,ml^{-1}$ Cholera Toxin (Sigma), $0.4\,\mu g\,ml^{-1}$ hydrocortisone (Sigma), $100\,mg\,l^{-1}$ penicillin and antibiotics as above, buffered to pH 7.4 with 5% $CO_2$, in air. Prior to use in assays, keratinocytes were exposed to the same medium as the other cell lines, for at least 48 h.

### *Binding studies and processing of EGF*

Binding studies were performed on confluent monolayers in 24 well culture dishes (Colstar), 2–5 days after seeding. Each well contained $1\text{–}5 \times 10^5$ cells depending upon the cell line or cell strain used. Cells were washed twice and incubated for 1 h in Hepes-buffered culture medium 199, containing 0.1% bovine serum albumin (BSA) prior to carrying out the binding assays. The binding was performed in 0.5 ml of medium 199 plus BSA. All assays were carried out in triplicate. For Scatchard analysis $^{125}$I-EGF was used over the range of 0.04 to 3.1 pmol. Nonspecific binding was measured in the presence of excess unlabelled EGF ($0.5\,\mu g\,ml^{-1}$). The nonspecific binding was consistently $<3\%$ of the total on the cell lines, and on the keratinocytes $<10\%$. At the completion of incubation, cells were washed 3 times in medium 199 plus 0.1% BSA, solubilised in 0.5% SDS in 1 M NaOH and their radioactivity determined. All experiments were performed at 4°C unless otherwise stated. Determination of numbers of binding sites and dissociation constants was by Scatchard analysis, and best fitting lines were determined by linear regression. Specific binding was determined by subtracting nonspecific binding from total bound. Free $^{125}$I-EGF was determined by subtracting the total bound from the amount added initially.

The effect of 2 mM chloroquine on the binding was determined over a six hour time course using the same protocol as described above.

### *Time course of binding at 4°C and 37°C*

Cells were incubated in medium 199 plus 0.1% BSA for 1 h and cooled to 4°C for subsequent measurements at the lower temperature. Binding assays were carried out as described above. $^{125}$I-EGF ($0.8 \times 10^6$ cpm/well, 0.88 pmol) in 0.5 ml medium 199/0.1% BSA was added to each well. Every fourth well also received $0.5\,\mu g$ unlabelled EGF. At the end of incubation for each time point triplicate cultures were washed, solubilised and counted.

### *Effects of chloroquine on processing of $^{125}$I-EGF at 37°C*

Chloroquine (Sigma) at a final concentration of 0.2 mM in medium 199 plus 0.1% BSA, was added to cells in 24 well culture dishes with $^{125}$I-EGF ($0.8 \times 10^6$ cpm/well, 0.88 pmol); controls omitted the chloroquine. Incubation was at 37°C, and triplicate cultures were washed, solubilised and counted over a 6-hour time course. Three wells of each cell type were counted at the end of the experiment. As an estimate of internalisation, separate identical cultures were incubated with 0.1% trypsin at 37°C for 5–10 min. Radioactive material released by trypsin and that remaining with the cells was determined.

*Measurement of degradation*

$^{125}$I-EGF (2.5 ml 1.1 pmol ml$^{-1}$; $1 \times 10^6$ cpm ml$^{-1}$) was incubated for 3 h at 37°C with LICR-LON-HN-5 cells, keratinocytes and SVK14 cells in 25 cm$^2$ flasks (Nunc). As a control, medium conditioned by exposure to a parallel flask of cells was incubated with $^{125}$I-EGF and treated similarly. HPLC chromatograms of 1 ml fractions of incubated medium were run on an ultrapore RPSC ($C_3$) column (Beckman Ltd. 7.5 cm × 4.6 mm i.d. 5 $\mu$ particle size). 0.155 NaCl (pH 2.1) was the primary solvent, acentonitrile was the secondary solvent. The following programme was used: 2.5 min with NaCl alone then a gradient of 2.0% acetonitrile min$^{-1}$ for 2.5 min; and 0.75% acetonitrile min$^{-1}$ thereafter, with a flow rate of 1 ml min$^{-1}$. One ml fractions were collected and counted on a gammacounter.

*Down regulation of EGF receptors*

Triplicate confluent multi-well cultures were grown for 24 h in DMEM, either with or without 50 ng ml$^{-1}$ EGF. After incubation, all cells were washed in medium 199/0.1% BSA and then incubated in medium 199/0.1% BSA (3 × 2 h) for a total of 6 h. Cell cultures were then cooled to 4°C and incubated for 4 h in 1 ml $^{125}$I-EGF ($1.7 \times 10^5$ cpm, 0.2 pmol) in medium 199/0.1% BSA. A fourth well also contained 0.5 $\mu$g of unlabelled EGF for measurement of non-specific binding. The amount of $^{125}$I-EGF bound specifically was determined as above.

### Results

Provisional studies comparing the binding of EGF at 37°C and 4°C in the squamous carcinoma cell lines and the SVK14 cells and normal keratinocytes (Figure 1) demonstrated an increased binding in the transformed cells at 37°C. Experiments involving incubation in the presence of chloroquine, a known inhibitor of EGF degradation (McKanna *et al.*, 1979), however, indicated that part of the binding seen at 37°C represented internalised EGF with subsequent degradation (Figure 2). Such degradation was confirmed by chromatography (Figure 3).

**Figure 1** Time course of binding of $^{125}$I-EGF at 4°C and 37°C. Each time point represents the mean value for 3 wells, minus non-specific binding in the presence of excess unlabelled EGF. In these experiments each well contained $5 \times 10^5$ normal keratinocytes (a) and $1.5 \times 10^5$ HN-5 tumour cells (b).

On the basis of these results all analyses of surface receptor density were carried out at 4°C when internalisation would be predicted to be inhibited (Willingham & Pastan, 1982), and thus the closest approximation to total surface receptor number could be made. With all cells examined at 4°C binding approached equilibrium after 4 h; these conditions were used for all subsequent assays.

Scatchard analysis (Figure 4) was carried out on the data. Normal keratinocytes, SVK14 cells and the tumour cell lines all had both high and low affinity receptors with dissociation constants in the range of $10^{-9}$ to $10^{-10}$ M (Table I). The tumour cells had 5–50 times the number of high and low affinity receptors of normal keratinocytes with LICR-LON-HN-5 cells having a total of $1.4 \times 10^7$ sites per cell.

It is unlikely that the observation of two different binding affinities present on each cell line was a result of differential iodination or oxidation of EGF during the labelling procedure, as a breast cell line, assayed for receptors, possessed a binding of single affinity using the same material (Cowley, 1984).

$^{125}$I-EGF is internalised and transported to lysosomes where it is degraded. Chloroquine inhibits

**Figure 2** Effects of chloroquine on processing $^{125}$I-EGF at 37°C. Controls omitted the chloroquine (broken line). In these experiments each well contained $2 \times 10^5$ SVK14 cells (a); $4 \times 10^5$ normal keratinocytes (b) and $1.4 \times 10^5$ HN-5 tumour cells (c).

**Figure 3** HPLC analysis of $^{125}$I-EGF degradation by HN-5 cells. One ml fractions of the HPLC eluate were counted and results are plotted as counts against time (min). The broken line represents the HPLC profile of $^{125}$I-EGF standard with a small free iodine peak after 3 min and the main peak eluting at 24 min. The solid line represents the profile after incubation with HN-5 tumour cells. The gradient in $CH_3CN$ applied to the column is shown (–·–·–).

this degradation and allows accumulation of the growth factor within the cells. In these experiments there is obviously accumulation of EGF in the presence of chloroquine demonstrating that the EGF does not only bind but is internalised. In HN-5 cells, the amount of trypsin-insensitive cell-associated radioactivity increased by 40% after chloroquine treatment, whilst the trypsin-sensitive binding increased by 10%. There is no obvious difference in the proportion of EGF accumulated in the presence of chloroquine in the tumour cells and transformed cells compared with normal keratinocytes.

HPLC analysis of $^{125}$I-EGF degradation by HN-5 cells is presented in Figure 3. A similar pattern of degradation was also seen with normal keratinocytes. No degradation occurred in conditioned medium in the absence of cells, demonstrating that degradation on EGF was a cell-dependent process.

Down regulation of receptors was demonstrated after pre-incubation overnight with unlabelled EGF (Table II). The degree of down regulation after 24 h was similar for the HN-5 cells, SVK14 and normal keratinocytes, showing a drop of 50–60% in the total amount of EGF specifically bound.

Thus all cell lines exhibited high and low affinity EGF receptors which were demonstrated to be down regulated by exogenous EGF in the case of the cell line HN-5, SVK14 cells and normal keratinocytes. The increased binding in the presence of chloroquine at 37°C and the effects of temperature supported the view that the binding seen at 37°C was a combination of surface binding, internalisation and degradation.

**Table II** Down regulation of EGF receptors

| | *Counts bound (cpm)* | | |
|---|---|---|---|
| *Cell line* | *with 50 ng EGF* | *without EGF* | *% Down regulation* |
| LICR-LON-HN-5 | 36,238 | 71,295 | 51.0% |
| SVK14 | 5,302 | 10,827 | 49.0% |
| Keratinocytes | 1,865 | 3,205 | 58.2% |

Each well contained $1.2 \times 10^5$ HN-5 cells, $2.1 \times 10^5$ SVK14 cells or $2.2 \times 10^5$ keratinocytes. The mean specific binding from 3 wells is shown.

**Figure 4** Binding of $^{125}$I-EGF to SVK14, keratinocytes, HN-5 and HN-10 cells. The standard binding assay was performed at 4°C as described previously. Each point represents the average of 3 wells minus non-specific binding. Results are srawn as Scatchard plots, allowing extrapolation to obtain numbers of receptors per cell and dissociation constant (See **Table I**). $1.4 \times 10^5$ SVK cells/well (a); $1.7 \times 10^5$ normal keratinocytes/well (b); $0.9 \times 10^5$ HN-5 from cells/well (c); $1.1 \times 10^5$ HN-10 tumour cells/well (d).

### Discussion

Original observations on the vulval epidermoid carcinoma cell line A431 (Fabricant *et al.*, 1977) demonstrated raised levels of EGF receptors ($2–3 \times 10^6$/cell) greater than any line previously reported. The gene for the receptor was subsequently found to be amplified and translocated (Schimizu *et al.*, 1984; Ullrich *et al.*, 1984).

The data presented here demonstrates that high levels of EGF receptor are a common feature of epidermoid carcinomas *in vitro*. Both carcinoma cell lines and simian virus transformed cells have similar capabilities to normal keratinocytes in terms of their ability to internalise, their sensitivity to chloroquine and their ability to degrade epidermal growth factor. The binding kinetics for all cells examined showed the typical pattern of a high and low affinity receptor with dissociation constants of $10^{-10}$ M and $10^{-9}$ M respectively.

Recent immunocytochemical techniques and a competitive binding assay utilising the monoclonal antibody EGFR-1 have shown that the increased receptor levels may be a constant feature of squamous carcinomas from the lung, head and neck, skin and cervix (Cowley *et al.*, 1984; Ozanne *et al.*, 1985). Concerning the mechanisms underlying the increased expression, studies on two of the cell lines described here show gene amplification and in another there is a specific translocation (Ozanne *et al.*, 1985). Studies on some breast cell lines have also demonstrated increased EGF receptor levels which are associated with amplification (Filmus *et al.*, 1985) and other tumours such as gliomas have been reported to have amplified genes (Libermann *et al.*, 1985).

EGF is known to be mitogenic on keratinocytes both *in vivo* and *in vitro*. In the case of epidermal carcinomas the high levels of EGF receptor could be envisaged as a potential mechanism whereby the tumours can respond to levels of EGF in the surrounding environment which are not mitogenic to normal cells. Support for this view is found with the epidermoid carcinoma cell line A431 which is stimulated to proliferate by levels of EGF which are insufficient to stimulate normal keratinocytes (Cowley *et al.*, 1984; Kawamoto *et al.*, 1983). The low levels of EGF required to stimulate proliferation could also be produced by partial occupancy of the receptor by a ligand produced by the cells such as an α-TGF like molecule thus producing a potentially autocrine mechanism (Sporn & Todaro, 1980).

The increased levels of receptor seen in the SV40 transformed cells may reflect their origin from a minor population of cells with high receptor levels combined with an inability of the cells to differentiate in culture. An alternative explanation is that the increased levels reflect the transformed phenotype of the cells as a stage in neoplastic progression.

There was no obvious correlation between the doubling times of the cell lines and the binding data (unpublished observation) supporting the view that the differences in receptors were due to an overall increase in receptor density rather than reflecting those cultures with a high proportion of cells in mitosis. There was also no correlation with anaplasia, the cell line with the highest levels, LICR-LON-HN-5 having been demonstrated to keratinise in culture with spinous cells, keratohyaline granule containing cells and the formation of a cornified envelope (Monaghan *et al.*, 1983; Knight *et al.*, 1984) while the line LICR-LON-HN-2 with ten-fold fewer receptors is relatively poorly differentiated both *in vitro* and *in vivo* (Easty *et al.*, 1981*a*; Cowley *et al.*, 1984).

Future analyses of the cell lines both at the molecular and the cell biological level will aid our understanding of both normal growth control and the aberrations that occur in malignancy. The data to date would suggest that increased levels of EGF receptor may well be an important component of the malignant phenotype in this cell type, but this is probably only one component of a complex picture of growth control in epithelial cells.

We would like to thank Drs M. Ellison and J. Ham who initiated these studies. We would like to aknowledge the donation of the SV40 transformed keratinocytes SVK14 by Dr Joyce Taylor-Papadimitriou of the Imperial Cancer Research Fund.

### References

ADAMSON, E.D. & REES, A.R. (1981). Epidermal growth factor receptors. *Mol. Cell Biochem.*, **34**, 129.

CARPENTER, G. & COHEN, S. (1979). Epidermal growth factor. *Ann. Rev. Biochem.*, **48**, 193.

COHEN, S. & ELLIOTT, G.A. (1963). The stimulation of epidermal keratinization by a protein isolated from the submaxillary gland of the mouse. *J. Invest. Dermatol.*, **40**, 1.

COWLEY, G., GUSTERSON, B. & KNIGHT, J. (1983). Growth in agar and tumour formation in immunologically incompetent mice as criteria for keratinocyte transformation. *Cancer Letters*, **21**, 95.

COWLEY, G., SMITH, J.A., GUSTERSON, B., HENDLER, F. & OZANNE, B. (1984). The amount of EGF receptor is elevated on squamous cell carcinomas. In *Cancer Cells*, Vol. I. The Tranformed Phenotype, p. 5. Cold Spring Harbor Labs.

COWLEY, G. (1984). *Markers of Malignant Transformation. Ph.D. Thesis.* University of London.

DAS, M., MIYAKAWA, T., FOX, C.F., PRUSS, R.M., AHARONOY, A. & HERSCHMAN, H.R. (1977). Specific radiolabelling of a cell surface receptor for epidermal growth factor. *Proc. Nat. Acad. Sci. USA*, **74**, 2790.

EASTY, D.M., EASTY, G.C., CARTER, R.L., MONAGHAN, P. & BUTLER, L.J. (1981*a*). Ten human carcinoma cell lines derived from squamous carcinomas of the head and neck. *Br. J. Cancer*, **43**, 772.

EASTY, D.M., EASTY, G.C., CARTER, R.L., MONAGHAN, P., PITMAN, M.R. & JAMES, T. (1981*b*). Five human tumour cell lines derived from a primary squamous carcinoma of the tongue, two subsequent local recurrences and two nodal metastases. *Br. J. Cancer*, **44**, 363.

FABRICANT, R.N., DELARCO, J.E. & TODARO, G.J. (1977). Nerve growth factor receptors on human melanoma cells in culture. *Proc. Nat. Acad. Sci. U.S.A.*, **74**, 565.

FILMUS, J., POLLAK, M.N., CAILLEAU, R. & BUICK, R.M. (1985). MDA-468, a human breast cancer cell line with a high number of epidermal growth factor (EGF) receptors, has an amplified EGF receptor gene and is growth inhibited by EGF. *Biochem. Biophys. Res. Comm.*, **128**, 898.

FITZPATRICK, S.L., BRIGHTWELL, J., WITTLIFF, J.L., BARROWS, G.H. & SCHULTZ, G.S. (1984). Epidermal growth factor binding by breast tumour biopsies and relationship to estrogen receptor and progestin receptor levels. *Cancer Res.*, **44**, 3448.

GILL, G.N., BERTICS, P.J., THOMPSON, D.M., WEBER, W. & COCHET, C. (1985). Structure and regulation of the epidermal growth factor receptor. In *Cancer Cells*, Vol. III. Growth Factors and Transformation, p. 11. Cold Spring Harbor Laboratory.

GOSPODAROWICZ, D., GREENBURG, G., BIALECKI, H. & ZETTER, B.R. (1978). Factors involved in the modulation of cell proliferation *in vivo* and *in vitro*: the role of fibroblasts and epidermal growth factors in the proliferative response of mammalian cells. *In Vitro*, **14**, 85.

GOSPODAROWICZ, D. (1981). Epidermal and nerve growth factors in mammalian development. *Ann. Rev. Physiol.*, **43**, 251.

GUSTERSON, B.A., COWLEY, G., SMITH, J.A. & OZANNE, B. (1984). Cellular localisation of human epidermal growth factor receptors. *Cell Biol. Int. Rep.*, **8**, 649.

HOLLENBERG, M.D. & CUATRECASAS, P. (1975). Epidermal growth factor: receptors in human fibroblasts and modulation of action by cholera toxin. *J. Biol. Chem.*, **250**, 3845.

HUNTER, T. & COOPER, J.A. (1981). Epidermal growth factor induces rapid tyrosine phosphorylation of proteins in A431 human tumour cells. *Cell*, **24**, 741.

KAWAMOTO, T., SATO, J.D., POLIKOFF, A. LE J., SATO, G.H. & MENDELSOHN, J. (1983). Growth stimulation of A431 cells by epidermal growth factor: identification of high affinity receptors for epidermal growth factor by an anti-receptor monoclonal antibody. *Proc. Natl. Acad. Sci. U.S.A.*, **80**, 1337.

KNIGHT, J., GUSTERSON, B.A., COWLEY, G. & MONAGHAN, P. (1984). Differentiation of normal and malignant human squamous epithelium *in vivo* and *in vitro*: a morphologic study. *Ultrastructural Path.*, **7**, 133.

KONDO, I. & SCHIMIZU, N. (1983). Mapping of the human gene for epidermal growth factor (EGFR) on the p13 leads to q22 region of chromosome 7. *Cytogenet. Cell Genet.*, **35**, 9.

KONTUREK, S.J., RADECKI, T., BRZOZOWSKI, I. & 6 others. (1981). Gastric cytoprotection by epidermal growth factor. *Gastroenterology*, **81**, 438.

LIBERMANN, T.A., NUSBAUM, H.R., RAZON, N. & 7 others. (1985). Amplification enhanced expression and possible rearrangement of EGF receptor gene in primary human brain tumour of glial origin. *Nature*, **313**, 144.

McKANNA, J.A., HAIGLER, H.T. & COHEN, S. (1979). Hormone receptor topology and dynamics: morphological analysis using ferritin-labelled epidermal growth factor. *Proc. Natl. Acad. Sci. U.S.A.*, **76**, 5689.

MONAGHAN, P., KNIGHT, J., COWLEY, G. & GUSTERSON, B. (1983). Differentiation of a squamous carcinoma cell line in culture and tumorigenicity in immunologically incompetent mice. *Virchows Arch. (Cell Pathol)*., **400**, 87.

OZANNE, B., SCHUM, A., RICHARDS, C.S. & 5 others. (1985). Evidence for increased EGF receptor in epidermal malignancies. In *Cancer Cell* Vol. III: Growth Factors and Transformation, p. 41. Cold Spring Habor Labs.

RHEINWALD, J.G. & GREEN, H. (1977). Tumorigenic keratinocyte lines requiring anchorage and fibroblast support cultured from human squamous cell carcinomas. *Nature*, **265**, 421.

SCHIMIZU, N., KONDO, I., GAMOU, S., BELINZADIN, M.A. & SCHIMIZU, Y. (1984). Genetic analysis of hyperproduction of epidermal growth factor receptors in human epidermoid carcinoma A431 cells. *Somatic Cell Mol. Genet.*, **10**, 45.

SMITH, J.A., HAM, J., WINSLOW, D.P., O'HARE, M.J. & RUDLAND, P.S. (1984). The use of HPLC in the isolation and characterization of mouse and rat epidermal growth factors and examination of apparent heterogeneity. *J. Chromatogr. Biomed. Appl.*, **305**, 295.

SPORN, M.B. & TODARO, G.J. (1980). Autocrine secretion and malignant transformation of cells. *New Engl. J. Med.*, **303**, 878.

TAYLOR-PAPADIMITRIOU, J., PURKIS, P., LANE, E.B., McKAY, I. & CHANG, S.E. (1982). Effects of SV40 transformation in the cytoskeleton and behaviour of human keratinocytes. *Cell Diff.*, **11**, 169.

ULLRICH, A., COUSSENS, J.S., HAYFLICK, J.S. & 12 others. (1984). Human epidermal growth factor receptor cDNA sequence and abberant expression of the amplified gene in A431 epidermoid carcinoma cells. *Nature*, **309**, 418.

WILLINGHAM, M.C. & PASTAN, I.H. (1982). Transit of epidermal growth factor through coated pits of the golgi system. *J. Cell Biol.*, **94**, 207.